# Antipsychotic treatment for epilepsy-related psychosis: hope from a newcomer

**DOI:** 10.1192/j.eurpsy.2025.2216

**Published:** 2025-08-26

**Authors:** V. Ferreira Amador

**Affiliations:** PSYCHIATRY, CENTRO HOSPITALARIO PADRE MENNI, SANTANDER, Spain

## Abstract

**Introduction:**

Psychosis has long been known to have an association with epilepsy. Slater’s 1963 descriptive case series provided the first modern definition of epilepsy-related psychosis, described as psychotic symptoms developing after the onset of epilepsy and occurring in clear consciousness, not exclusively during or right after a seizure. Up to 7% of individuals with epilepsy have a co-morbid psychotic illness. Consensus guidelines by the International League of Epilepsy recommend treating epilepsy-related psychosis similarly to other categories of psychosis. Despite the widespread consensus that the prescription of an antipsychotic is possible without risk in an epileptic patient treated with AEDs, there have always been special considerations about prescribing antipsychotics for seizure-related psychosis. Furthermore, a review article by Adachi et al. specifically warns that concerns regarding seizure threshold can lead to the undertreatment of this particular type of psychosis.

**Objectives:**

Case report.

**Methods:**

Case report.

**Results:**

**Case presentation:** Our patient has generalized epilepsy, with several incomes due to generalized tonic-clonic seizure and mild diffuse encephalopathy since childhood. By the age of 15 years, he started developing suspicion, self-reference, persecutory and damage delusions, visual hallucinations and episodes of aggressiveness towards people surrounding him. He had been treated with multiple antiepileptics and antipsychotics drugs, without achieving a long-term stabilisation until now. While the seizures were controlled in his early ages; ever since the onset of the psychosis both the psychotic symptoms and the seizures got impossible to handle, mainly because of his lack of tolerance to almost every antipsychotic used. Our primary hypothesis is that these antipsychotics lowered the seizure threshold, triggering new episodes of tonic-clonic seizures and arousing the patient’s desire of abandon the treatment -the latter has been confirmed by the patient-. At this point, we considered the recently released brexpiprazol as a potentially efficient treatment. After a proper explanation of our clinical reasons to prescribe this new drug, especially its unique pharmacodynamics, we adquired the patient’s consent to start with it. So far, the response is being excellent, which is particularly impressive given the fact that he is taking the maximum daily dose of brexpiprazol (4mg). Not only the psychotic symptoms are restrained, but also he has not experienced any seizure since the introduction. As a key outcome, the patient has developed a significant insight regarding his condition and the need of treatment.

**Conclusions:**

The treatment of epilepsy-related psychosis is a really delicate subject. Brexpiprazol is a modern drug, with an innovative pharmacodynamics and an excellent side-effect profile, and should be taken into consideration in every patient with seizure-related psychosis.

**Disclosure of Interest:**

None Declared

